# Brewing-Method-Dependent Changes in the Bioactive Compound Profile and Antioxidant Potential of Coffee Beverages

**DOI:** 10.3390/molecules31122163

**Published:** 2026-06-19

**Authors:** Magdalena Sęk, Urszula Cięciel, Małgorzata Tkacz, Sascha Rohn, Michał Halagarda

**Affiliations:** 1 Department of Food Product Quality, Krakow University of Economics, ul. Sienkiewicza 5, 30-033 Krakow, Poland; magdalena.sek@uek.krakow.pl (M.S.);; 2 Department of Food Chemistry and Analysis, Institute of Food Technology and Food Chemistry, Technische Universität Berlin, Kaiserin-Augsta-Allee 14, 10553 Berlin, Germany; rohn@tu-berlin.de

**Keywords:** coffee, chlorogenic acids, antioxidants, HPLC-online TEAC

## Abstract

Coffee is an important dietary source of bioactive antioxidant compounds contributing to the antioxidant properties of coffee beverages. While brewing affects yield of total antioxidants, it is still not really clear which individual (phenolic) compounds contribute to the antioxidant activity the most. A method combining chromatographic separation and individual antioxidant evaluation might therefore be useful. This study aimed at evaluating the antioxidant potential of the compounds in coffee beverages using a high-performance liquid chromatography approach directly coupled to the well-known trolox equivalent antioxidative capacity (TEAC) assay (HPLC-onlineTEAC). The study further evaluated the influence of different brewing methods (‘Americano’, ‘V60’, ‘French press’, and ‘cold brew’) on the bioactive compound profile and antioxidant potential of Arabica coffee beverages. The brewing method significantly affected caffeine content, chlorogenic acid composition, total phenolic content (TPC), and antioxidant activity of the analyzed beverages (*p* < 0.05). Cold brew samples exhibited the highest total radical scavenging activity and concentrations of major caffeoylquinic acid isomers (3-CQA, 4-CQA, and 5-CQA). In contrast, “French-pressed” beverages were characterized by the highest TPC values, while V60 samples generally showed the lowest antioxidant-related parameters. Chlorogenic acids accounted for more than 84% of the total antioxidant potential of all analyzed beverages, whereas monocaffeoylquinic acids represented the dominant fraction responsible for radical-scavenging activity. The results indicate that prolonged low-temperature extraction favors the recovery and preservation of highly reactive chlorogenic acid isomers and contributes to the enhanced antioxidant potential of coffee beverages, beyond the effect of coffee dose alone.

## 1. Introduction

Coffee is one of the most widely consumed beverages worldwide [1,2] and constitutes an important source of bioactive compounds with antioxidant properties capable of neutralizing free radicals [3]. After more than twenty years of research, it is apparently textbook knowledge that excessive production of reactive oxygen and nitrogen species leads to oxidative stress, which is recognized as one of the key mechanisms underlying the pathogenesis of cardiovascular, neurodegenerative, and neoplastic diseases [4,5,6]. Moreover, dietary intervention with antioxidants might moderate oxidative stress to a certain extent [7]. However, optimum levels are still not defined comprehensively, as many individual factors might affect oxidative status of a human organism [8,9]. Anyway, it seems to be promising that consumers might be able to vary their antioxidant intake. Because of significant consumption, antioxidant properties of coffee are considered one of the principal determinants of its potential chemopreventive effects [10,11].

The antioxidant properties of coffee are primarily attributed to the presence of individual phenolic compounds, particularly chlorogenic acids (CGAs) and their derivatives, which constitute the dominant phenolic fraction of coffee beans [12,13,14]. Another important constituent of coffee is caffeine, which, despite exhibiting weaker antioxidant activity than polyphenols, may contribute to the overall antioxidant potential of the beverage due to its high concentration in coffee and its presence within a complex matrix of bioactive compounds [15,16,17].

The chemical composition and functional properties of coffee infusions are strongly influenced by the coffee species, geographical origin, roasting degree, and extraction parameters, including brewing temperature, extraction time, grind size, and beverage preparation method. It has already been demonstrated that different brewing techniques, including Americano, pour-over methods (e.g., V60), French press, and low-temperature extraction (cold brew), result in significant differences in the content of phenolic compounds, caffeine, and the overall antioxidant activity of coffee beverages [18,19,20]. High-temperature brewing methods, such as Americano and pour-over techniques (e.g., V60), generally enable the production of beverages characterized by high concentrations of bioactive compounds, including CGAs, per unit volume despite relatively short extraction times. This effect is associated with the application of high pressure and fine grinding of coffee beans, both of which enhance the extraction efficiency of soluble compounds [21,22,23]. In contrast, cold brew beverages are prepared under conditions of prolonged low-temperature extraction, which affects the release kinetics of bioactive compounds. However, the available literature data regarding the CGA content and antioxidant activity of cold brew beverages remain inconclusive and appear to depend on extraction parameters, roasting degree, and the analytical methods applied [19,24,25,26].

It should be emphasized, however, that the vast majority of previous studies have focused on the determination of total phenolic content, caffeine concentration, or the total antioxidant activity of coffee beverages, typically assessed using spectrophotometric assays such as the 2,2-diphenyl-1-picrylhydrazyl (DPPH) radical scavenging assay, 2,2′-azinobis-(3-ethylbenzothiazoline-6-sulphonate) (ABTS) radical cation decolorization assay, and the ferric reduction antioxidant potential (FRAP) assay [16,19,23,26,27,28,29]. Although these methods provide valuable information regarding the overall antioxidant potential of coffee infusions, they do not allow for the determination of the individual contribution of bioactive compounds with regard to free radical scavenging reactions, nor do they enable the assessment of their individual reactivity within the complex composition and matrix effects of the beverage. Especially the latter are prone to be influenced by the brewing method, as more or less lipids and complex carbohydrates, including melanoidins, form the Maillard reaction, melanin- and melanoidin-like compounds are extracted, all of which are capable of reacting in redox reactions [30].

In recent years, increasing attention has been directed toward analytical techniques that enable the assessment of the selective reactivity of individual compounds towards free radicals. The coupling of high-performance liquid chromatography (HPLC) with online radical scavenging assays enables the direct observation of changes in chromatographic profiles following the reaction between antioxidants and free radicals, thereby allowing the identification of compounds that actively participate in antioxidant processes [31,32,33,34,35,36]. This approach extends beyond conventional quantitative methods and provides insight into the functional activity of individual compounds within complex matrices [37], such as coffee infusions.

Despite the significant number of studies investigating the influence of brewing methods on the antioxidant properties of coffee beverages, there remains a lack of research focused on the comparative evaluation of the actual reactivity of individual antioxidants present in infusions prepared using different techniques, including Americano, pour-over methods, and cold brew extraction. Therefore, the present study aimed to test an HPLC online TEAC detection system for comparing the antioxidant potential of coffee beverages prepared using different extraction methods and to evaluate the contribution of CGAs, including the major caffeoylquinic acid isomers (3-CQA, 4-CQA, and 5-CQA), to the free radical scavenging activity of the analyzed infusions.

## 2. Results and Discussion

The available literature data indicate that the coffee brewing method may significantly affect the chemical composition of the finally consumed beverage, thereby modifying its antioxidant properties and the profile of bioactive compounds present in the infusion [21,25,26,38,39]. The results obtained in the present study (Table 1) also confirm that the extraction method significantly affected acidity, caffeine content, radical scavenging activity, and total phenolic content (TPC) of the analyzed coffee beverages.

**Figure 1:**
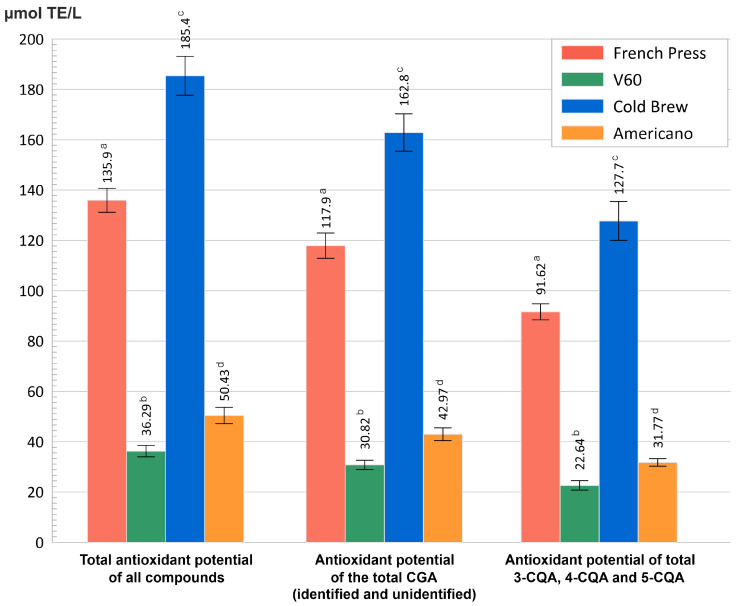
Antioxidant activity of total compounds, total chlorogenic acids, and major caffeoylquinic acids in coffee beverages depending on the different brewing methods, expressed as µmol of trolox equivalent per liter of beverage (μmol TE/L). Different superscript letters within the same compound indicate statistically significant differences (*p* < 0.05).

**Figure 2:**
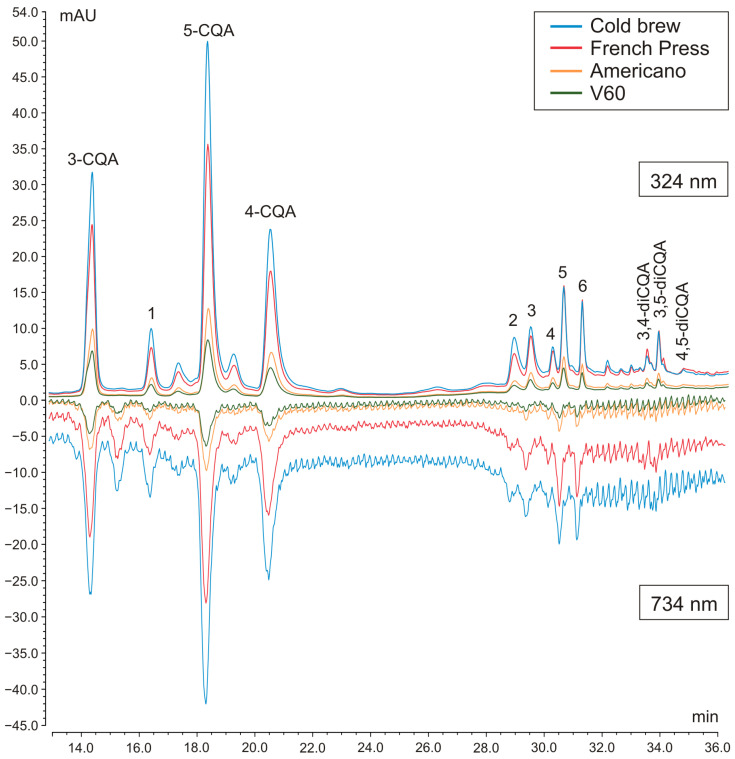
HPLC-online TEAC chromatograms showing detected identified and unidentified (1–6) chlorogenic acids (positive peaks) and their corresponding antioxidant response (negative peaks).

Significant differences were also observed (Figure 1) in the total antioxidant potential of all detected compounds, and the antioxidant potential specifically associated with the major caffeoylquinic acid isomers (3-CQA, 4-CQA, and 5-CQA), as determined by a high-performance liquid chromatography approach directly coupled to the well-known trolox-equivalent antioxidative capacity (TEAC) assay (HPLC-onlineTEAC). These findings are illustrated in the chromatograms presented in Figure 2, where positive peaks represent the detected compounds and their relative abundances, whereas the corresponding negative peaks reflect their antioxidant activities measured by the online TEAC detection system.

### 2.1. Effect of Brewing Method on the Physicochemical Properties and Bioactive Compound Profile of Coffee Beverages

Among all analyzed brewing methods, cold brew samples were characterized by the highest total acidity and caffeine content, followed by French press, Americano, and V60 beverages. The caffeine concentration in the cold brew infusion reached 939.28 mg/L, whereas in the V60 coffee it was only 217.57 mg/L. However, when the caffeine content was expressed per gram of ground coffee used for brewing (Supplementary Table S1), French press beverages exhibited significantly higher values than cold brew samples. This finding suggests that the elevated caffeine concentrations observed in cold brew beverages were influenced not only by the extraction conditions but also by the larger amount of coffee used during beverage preparation. Nevertheless, the prolonged extraction time characteristic of cold brew, which enables more efficient diffusion of water-soluble alkaloids despite the lower extraction temperature applied, may still contribute to efficient caffeine recovery into the final beverage. Similar relationships were reported by Rao and Fuller [26], who demonstrated that an extended duration of extraction, as in the cold brew method, promotes high caffeine extraction efficiency.

Significant differences were also observed in the TPC. The highest TPC values were obtained for beverages prepared using the French press method (1040 mg GAE/L), followed by cold brew (753 mg GAE/L), V60 (655 mg GAE/L), and Americano (621 mg GAE/L). Normalization of TPC values to the mass of coffee used for brewing also altered the ranking of the analyzed brewing methods (Supplementary Table S1). Americano beverages exhibited the highest TPC expressed per gram of coffee, followed by French press, V60, and cold brew samples. These results indicate that the total phenolic content measured in the beverage is influenced by brewing conditions, filtration type and coffee dose applied during preparation. The highest total phenolic content, expressed per gram of coffee used, observed in Americano beverages may be associated with the high extraction efficiency characteristic of the espresso brewing process, which serves as the basis for this beverage. The high pressure and temperature applied during extraction promote the release of soluble compounds, including chlorogenic acids and other phenolic constituents [23]. Furthermore, Caprioli et al. [22] demonstrated that espresso machine operating parameters, particularly pressure and temperature, significantly influence the extraction efficiency of chlorogenic acids. These findings therefore suggest that the Americano brewing method enables efficient recovery of phenolic compounds per unit of coffee material. The lower TPC observed when expressed per liter of the final beverage is most likely attributable to the subsequent dilution of espresso with water rather than to a lower efficiency of the extraction process itself. The high TPC observed in French press samples may be associated with the absence of paper filtration, which promotes the presence of a more pronounced amount of suspended particles and larger compounds such as coffee melanins, melanoidins, and high-molecular-weight phenolic compounds in the beverage. Similar observations were reported by Olechno et al. [40], Derossi et al. [41], and Ludwig et al. [23], who demonstrated that the brewing method, grind size, and water–coffee contact time influence the extraction of phenolic compounds and the antioxidant activity of coffee beverages. Similar relationships have also been observed in tea infusions. Pastoriza et al. [42] demonstrated that infusions prepared from tea bags containing more finely fragmented plant material were characterized by higher polyphenol content and greater antioxidant activity than infusions brewed from whole tea leaves. The authors attributed this effect to the larger surface area available for contact with water and the more efficient extraction of bioactive compounds. These findings suggest that, in both tea and coffee, the physical properties of the raw material as well as filtration conditions may significantly influence the recovery of phenolic compounds.

**Figure 3:**
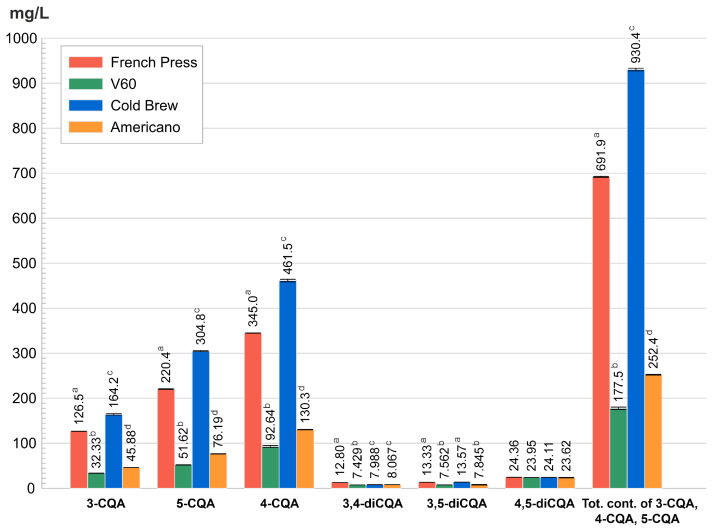
Concentrations of identified caffeoylquinic and dicaffeoylquinic acids (mg/L) in coffee beverages prepared using different brewing methods. Different superscript letters within the same compound indicate statistically significant differences (*p* < 0.05).

Among the quantified CGAs, the highest concentrations were observed for 5-CQA, which constituted the dominant isomer in all analyzed coffee beverages (Figure 3), in agreement with the findings reported by Jeon et al. [21]. The highest concentrations of 3-CQA, 4-CQA, and 5-CQA were determined in cold brew samples. Particularly high levels were recorded for 5-CQA, whose concentration in the cold brew beverage reached 305 mg/L, whereas in the V60 beverage it was only 52 mg/L. High concentrations of CGAs were also observed in French press beverages, which is consistent with observations reported by Moeenfard et al. [43]. Moreover, while Angeloni et al. [20] did not report significant differences in dicaffeoylquinic acid (diCQA) isomer content among French press, V60, and cold brew beverages, the present study demonstrated that French press brews exhibited the highest concentrations of 3,4-diCQA and 3,5-diCQA, suggesting that this brewing method may particularly favor the extraction of diCQA isomers.

**Figure 4:**
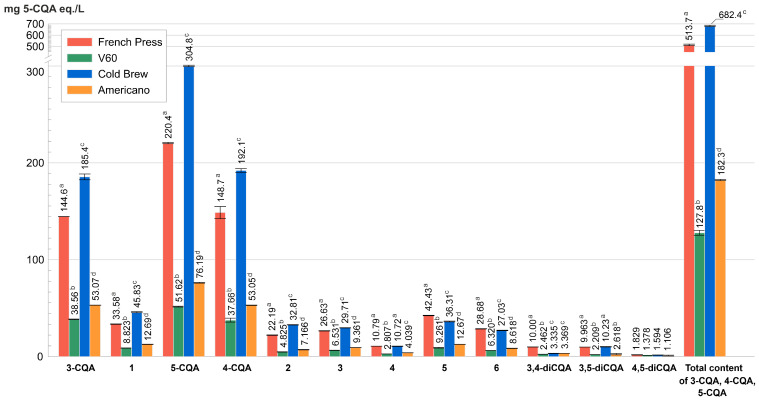
Contents of identified and unidentified (1–6) chlorogenic acids expressed as 5-CQA equivalents (mg 5-CQA eq./L) in coffee beverages prepared by different brewing methods. Different superscript letters within the same compound indicate statistically significant differences (*p* < 0.05).

Moreover, as illustrated in Figure 4, the inclusion of both identified and unidentified compounds, expressed as 5-CQA equivalents, allowed for a more comprehensive comparison of the extraction methods. The observed differences among brewing techniques indicate that extraction conditions affect not only the concentrations of identified CGA isomers but also the abundance of other, as yet unidentified, compounds detected in the analyzed beverages. These findings confirm that the extraction of CGAs and related phenolic constituents is strongly dependent on brewing conditions. Similarly, Moeenfard et al. [43] demonstrated that the brewing technique significantly affects the content of the major CQA isomers in coffee beverages and that their total concentration may vary considerably depending on the extraction method applied.

Importantly, normalization of the results to the mass of ground coffee used for brewing did not substantially alter the overall trends observed for chlorogenic acids (Supplementary Table S1). Cold brew beverages remained characterized by the highest contents of 4-CQA, 5-CQA, and total CQA expressed per gram of coffee, whereas French press brews exhibited higher contents of 3-CQA. These findings suggest that the elevated chlorogenic acid concentrations observed in cold brew beverages cannot be attributed solely to differences in coffee dose but are also strongly influenced by the extraction conditions associated with this brewing method. The high CGA content observed in cold brew samples is most likely associated with the combination of prolonged extraction time and reduced thermal degradation of these compounds. However, it should also be noted that the ranking of brewing methods for dicaffeoylquinic acid isomers changed after normalization, with Americano beverages exhibiting the highest contents of 3,4-diCQA and 4,5-diCQA, while both Americano and French press brews were characterized by the highest levels of 3,5-diCQA. As reported by Farah and Donangelo [14], CGAs undergo numerous chemical transformations during coffee processing (especially fermentation and roasting), including polymerization, isomerization, but also hydrolysis and partial degradation reactions. The roasting process also promotes the formation of CGA lactones and melanoidins, which may contribute to the antioxidant activity of coffee beverages [41]. Therefore, extraction conducted at lower temperatures may favor the preservation of some of the thermolabile phenolic compounds in the final beverage. However, an indirect effect might also be possible. The high temperature impact (during roasting) might also lead to radical-mediated reactions (such as in the Maillard reaction), already consuming and transforming phenolic compounds [44]. Similar relationships have also been observed in tea infusions. Studies have shown that extending the contact time between plant material and water may increase the recovery of phenolic compounds even at lower extraction temperatures [42]. In turn, Vuong et al. [45] indicated that the efficiency of catechin extraction from tea depends primarily on extraction time, temperature, and extraction conditions. These findings are consistent with the high chlorogenic acid content observed in cold brew beverages in the present study, suggesting that prolonged contact between water and plant material may partially compensate for the lower extraction temperature.

At the same time, it should be emphasized that the available literature data concerning cold brew beverages remain inconclusive. Herawati et al. [38] demonstrated that beverages prepared using the V60 method contained higher concentrations of CQA isomers and caffeine than cold brew samples. Those authors suggested that these differences could be attributed, among other factors, to variations in the coffee-to-water ratio, extraction time, and the dynamics of water–coffee contact applied for the respective brewing methods. This suggests that the final profile of bioactive compounds depends not only on the brewing technique itself but also on the overall set of extraction parameters applied.

### 2.2. Effect of Brewing Method on the Antioxidant Activity and Chlorogenic Acid Contribution to the Antioxidant Potential of Coffee Beverages

Also in the present study, the brewing method significantly affected the antioxidant activity of the analyzed coffee beverages (*p* < 0.001) (Table 1 and Figure 1). The highest DPPH radical scavenging activity was observed for cold brew samples (30.74%), followed by French press (19.85%), Americano (16.02%), and V60 beverages (15.31%). The obtained results indicate that extraction conditions affect not only the amount of extracted bioactive compounds but also their functional properties. The highest antioxidant activity observed in cold brew beverages may be associated with the high concentration of CGA present in these samples. CGA are considered among the principal antioxidants in coffee due to their ability to neutralize free radicals, which is supported by numerous studies demonstrating a relationship between CGA content and the antioxidant activity of coffee beverages [14,16,23,25]. The present findings are consistent with observations reported for tea infusions, where antioxidant activity was shown to depend not only on the amount of extracted phenolic compounds but also on their qualitative profile and the transformations occurring during the extraction process [42]. These results suggest that the free radical scavenging capacity of coffee beverages may likewise be influenced not only by the total content of polyphenols but also by the composition of bioactive compounds and their individual antioxidant properties.

The analysis of the antioxidant potential of individual compounds using the HPLC-onlineTEAC approach demonstrated that the highest total antioxidant potential of all detected compounds was observed in cold brew samples (185 µmol TE/L), followed by French press (136 µmol TE/L), Americano (50.4 µmol TE/L), and V60 beverages (36.3 µmol TE/L). These findings indicate that the compounds present in cold brew beverages exhibited the most intense capacity to scavenge ABTS radicals among all analyzed brewing methods. However, when the antioxidant potential was expressed per gram of coffee used for brewing (Supplementary Table S1), cold brew and French press beverages exhibited comparable values, both remaining significantly higher than those observed for Americano and V60 samples. This finding suggests that differences in coffee dose partially contributed to the antioxidant potential measured in the final beverages, although the extraction conditions associated with cold brew and French press preparation still promoted the recovery of compounds exhibiting high radical scavenging activity.

From a consumer perspective, however, these differences may not necessarily translate into meaningful differences in dietary exposure to coffee-derived bioactive compounds. Although cold brew beverages exhibited the highest chlorogenic acid content and antioxidant potential, the choice of a coffee brewing method is influenced not only by its potential health benefits but also by sensory characteristics, convenience, habitual consumption patterns, and individual preferences. Consequently, the actual intake of coffee-derived bioactive compounds depends both on the composition of the beverage and the frequency with which it is consumed. This is particularly important given that brewing methods associated with a higher antioxidant potential are not necessarily the most commonly selected by consumers. Czarniecka-Skubina et al. [46], in a survey involving 1500 Polish respondents, reported that espresso-based methods and V60 were among the most frequently declared brewing techniques (77.7% and 61.5% of responses, respectively), whereas French press and cold brew represented relatively niche choices, indicated by only 15.4% and 12.9% of respondents, respectively. Therefore, despite the favorable antioxidant characteristics observed for cold brew beverages in the present study, their current contribution to the dietary intake of chlorogenic acids at the population level may remain limited. However, recent market trends indicate a growing interest in cold brew coffee [47], suggesting that its contribution to dietary intake of coffee-derived bioactive compounds may become more relevant in future consumption patterns.

As indicated in Table 2, a particularly important contribution was attributed to the CGA isomers, whose total antioxidant potential accounted for 84.92–87.83% of the total antioxidant potential of all analyzed compounds, depending on the brewing method applied. These results indicate that CGA isomers constituted the dominant group of compounds responsible for the antioxidant properties of the analyzed coffee beverages. The highest antioxidant potential of all quantified CGA isomers was obtained for cold brew samples (163 µmol TE/L), followed by French press (118 µmol TE/L), Americano (43 µmol TE/L), and V60 beverages (30.8 µmol TE/L). A similar pattern was observed after normalization to the mass of coffee used for brewing (Supplementary Table S1). The total antioxidant potential of CGAs remained significantly higher in cold brew and French press beverages than in Americano and V60 samples. A particularly high contribution to the total antioxidant activity was observed for the individual CGA isomers, namely 3-CQA, 4-CQA, and 5-CQA. The combined antioxidant potential of these three compounds accounted for 62.39–68.89% of the total antioxidant potential of the beverages and for 73.46–78.44% of the activity of all quantified CGAs. This indicates that the majority of the free radical scavenging capacity attributed to the CGA fraction resulted primarily from the presence of mono-CQAs. The obtained results therefore suggest that not all CGAs exhibit comparable reactivity toward free radicals and that the actual antioxidant potential of coffee beverages depends not only on the TPC but also on the qualitative profile of individual isomers.

**Figure 5:**
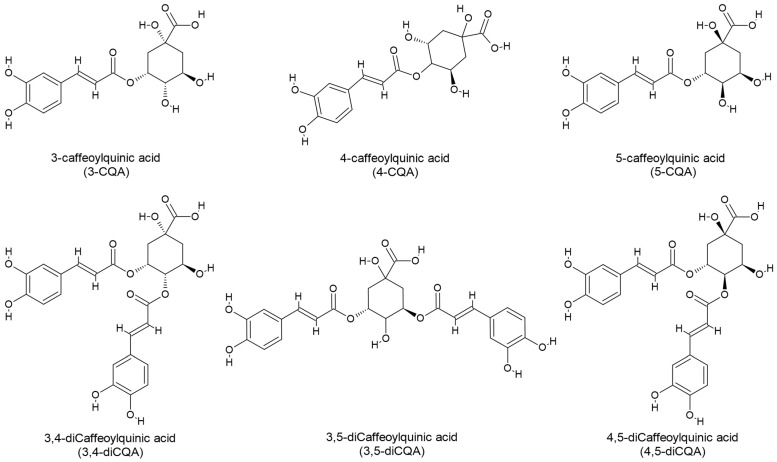
Chemical structures of quantified chlorogenic acids (CQA and diCQA).

The highest contribution of CQA isomers was observed in cold brew samples, which may suggest that prolonged extraction performed at low temperature favors the preservation of the isomers exhibiting the highest antioxidant reactivity. These findings are consistent with the observations reported by Jeon et al. [21] and Liang and Kitts [16], who indicated that CGA isomers are characterized by a high free radical scavenging capacity resulting from the presence of hydroxyl groups capable of efficiently donating hydrogen atoms (see Figure 5).

The lower antioxidant activity observed in V60 beverages may be associated both with the lower CGA content and with the use of paper filtration, which limits the transfer of certain phenolic compounds and other antioxidant-active constituents into the beverage. Similar observations were reported by Derossi et al. [41] and Ludwig et al. [23], who emphasized the significant influence of the filtration method on the extraction efficiency of antioxidants.

It is also worth emphasizing that the highest TPC did not necessarily correspond to the highest antioxidant activity. Although French press beverages were characterized by the highest TPC values, the highest DPPH scavenging activity was observed for cold brew samples. This may result from differences in the qualitative profile of phenolic compounds, their mutual interactions, and their reactivity toward free radicals. Furthermore, the Folin–Ciocalteu assay determines the overall reducing capacity of the sample rather than exclusively the content of biologically active polyphenols, which may lead to an overestimation of TPC values in the presence of other reducing compounds, such as some melanoidins [48,49].

These findings also confirm the suitability of the HPLC-online TEAC technique, which enables the evaluation of which individual compounds are present and responsible within the complex beverage matrix and exhibit actual reactivity toward free radicals. In contrast to conventional spectrophotometric methods, such as TPC determination or total DPPH scavenging activity, this technique makes it possible to identify which specific compounds are responsible for radical neutralization and to determine their actual contribution to the total antioxidant potential of the beverage. Similar conclusions were already presented by Li et al. [31], Qian et al. [32] and Yan et al. [34], who emphasized that the combination of liquid chromatography with radical scavenging reactions enables a more comprehensive assessment of the antioxidant activity of complex natural matrices.

### 2.3. Principal Component Analysis of Coffee Beverages Prepared Using Different Brewing Methods

Principal component analysis (PCA) was performed to provide an integrated overview of the relationships among the analyzed variables and to visualize similarities and differences between coffee samples prepared using different brewing methods. The first two principal components accounted for 97.06% of the total variance, indicating that the PCA model effectively summarized the variability of the dataset. The first principal component (PC1) explained 85.82% of the total variance, whereas the second principal component (PC2) accounted for 11.25%.

**Figure 6:**
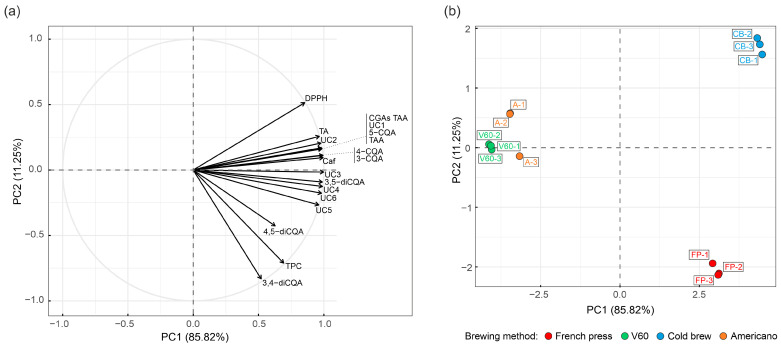
Principal component analysis (PCA) of coffee samples prepared using different brewing methods. (**a**) Variable factor map; (**b**) individual factor map.

The variables factor map (Figure 6a) confirmed that the majority of physicochemical and antioxidant-related variables were positively associated with PC1, supporting the overall compositional differences observed between these brewing methods. The individuals factor map (Figure 6b) revealed a clear separation of the samples according to brewing method along PC1. Cold brew and French press samples were located on the positive side of PC1, whereas V60 and Americano samples were grouped on the negative side. This distribution indicates that cold brew and French press coffees were characterized by generally higher values of most analyzed parameters compared to V60 and Americano samples.

Furthermore, PC2 enabled differentiation between cold brew and French press samples. Cold brew samples were associated with higher DPPH radical scavenging activity, whereas French press samples were more strongly correlated with higher TPC values and diCQA isomers, particularly 3,4-diCQA and, to a lesser extent, 4,5-diCQA. Although statistically significant differences in 4,5-diCQA content were not observed among the brewing methods, its contribution to the PCA model suggests that this compound may still participate in the multivariate differentiation of the beverages. Similarly, 3,5-diCQA was positioned on the same side of the PCA plot as the French press samples, despite the lack of significant differences between French press and cold brew beverages observed in the univariate analysis. This indicates that PCA captured broader compositional relationships among the beverages, in which diCQA isomers contributed to the separation of French press brews from the remaining brewing methods. Therefore, the PCA model further supports the observation that the French press brewing method may favor the extraction of diCQA isomers. Overall, these findings indicate that, although cold brew and French press brewing methods yielded extracts rich in bioactive compounds, they differed in the dominant antioxidant-related characteristics and phenolic composition.

## 3. Materials and Methods

### 3.1. Preparation of Coffee Brews

Dark-roasted Arabica coffee beans (100% *Coffea arabica*) of Brazilian origin were obtained from a local coffee roastery in Krakow, Poland. Prior to brewing, the coffee beans were ground using a Mahlkönig EK43 burr grinder (Mahlkönig GmbH & Co. KG, Hamburg, Germany) adjusted to obtain a uniform medium grind size, thereby minimizing variability in extraction associated with differences in particle size distribution. The ground coffee was used to prepare four types of coffee beverages: Americano (A), French press (FP), V60, and cold brew (CB). Brewing parameters were selected to reflect conditions commonly applied to the respective preparation methods based on previously published studies [20,21,23,50,51]. The study was designed to evaluate the influence of brewing techniques on the chemical composition and antioxidant properties of coffee beverages under standardized preparation conditions rather than to assess differences associated with coffee origin or raw material characteristics.

Americano. Americano coffee was prepared using a De’Longhi Dinamica automatic espresso machine (De’Longhi, Treviso, Italy). Briefly, 6.5 g of ground coffee was placed in the coffee machine container and extracted with 25 mL of hot water over 30 s. The obtained espresso was subsequently diluted with hot water (96 °C) to a final volume of 200 mL.

French press. French press coffee was prepared using a cylindrical glass vessel equipped with a plunger and a metal mesh filter (Bialetti, Coccaglio, Italy). Extraction was carried out using 40 g of ground coffee and 700 mL of hot water (90 °C). After 4 min of extraction, the plunger was slowly pressed to separate the liquid coffee extract from the coffee grounds.

V60. Coffee prepared using the V60 method employed a cone-shaped dripper with a paper filter and a glass server (Hario, Tokyo, Japan). Prior to extraction, the paper filter was rinsed with a small amount of hot water. Subsequently, 10 g of ground coffee was placed in the dripper and 30 mL of hot water (95 °C) was added to initiate the pre-infusion step (“blooming”). After 30 s, additional portions of hot water were poured in circular motions in two stages to obtain a final beverage volume of 200 mL within 2 min.

Cold brew. Cold brew coffee was prepared using a cold brew coffee pot (Mizudashi, Hario, Japan) consisting of a glass vessel and a polyester mesh filter with a lid. A total of 60 g of ground coffee was immersed in 800 mL of water at 25 °C and extracted under refrigerated conditions at 7 °C for 12 h. After extraction, the coffee beverage was separated from the coffee grounds.

### 3.2. Titratable Acidity

Titratable acidity was determined according to AOAC procedures [52], using a SevenCompact digital pH meter equipped with an InLab Expert Pro-ISM electrode (both Mettler Toledo, Greifensee, Switzerland). Briefly, 50 mL of coffee infusion was titrated with 0.1 M NaOH under constant stirring until the endpoint of pH 8.2 was reached.

### 3.3. DPPH Radical Scavenging Assay

The ability of coffee samples to scavenge the 1,1-diphenyl-2-picrylhydrazyl (DPPH) radical was determined according to the procedure described by Vignoli et al. [49]. Briefly, 10 µL of coffee infusion was added to a reaction mixture consisting of 1 mL of 100 mM acetate buffer (pH 5.5), 1 mL of ethanol, and 0.5 mL of a 250 µM ethanolic DPPH radical solution. Following incubation for 10 min, the absorbance of the solution was measured at 517 nm using a Nanocolor UV/VIS II spectrophotometer (Macherey-Nagel GmbH & Co. KG, Düren, Germany). The control sample was prepared in the same manner without the addition of coffee infusion, whereas the blank sample consisted of the reaction mixture without the DPPH radical solution. Antioxidant activity was expressed as the percentage of radical scavenging activity and calculated according to the following equation:

(1) $$\%\;radical\;scavenging\;activity=\frac{Abs\;control-Abs\;sample}{Abs\;control}\times 100\%$$

### 3.4. Total Polyphenol Content (TPC)

The total polyphenol content was determined using the Folin–Ciocalteu method according to Vignoli et al. [49]. Briefly, 0.1 mL of coffee infusion was diluted with deionized water to a volume of 7.5 mL. Subsequently, 0.3 mL of 0.9 mol/L Folin–Ciocalteu reagent and 1 mL of 20% sodium carbonate (Na_2_CO_3_) solution were added. The reaction mixture was then adjusted to a final volume of 10 mL with deionized water and incubated at room temperature for 60 min prior to absorbance measurement at 765 nm using a Nanocolor UV/VIS II spectrophotometer (Macherey-Nagel GmbH & Co. KG, Düren, Germany). The results were expressed as gallic acid equivalents (mg GAE/L).

### 3.5. Chlorogenic Acid Content and Antioxidant Activity

The profile of CGA and the antioxidant activity of coffee extracts were determined using the high-performance liquid chromatography–online trolox equivalent antioxidant capacity (HPLC–online TEAC) method, which enables the simultaneous identification of individual compounds and evaluation of their antioxidant activity [53].

Prior to chromatographic analysis, brewed coffee samples were appropriately diluted and supplemented with 10 µL of a 10 mM trolox solution as an internal standard. Before injection, the samples were filtered through 0.22 µm polytetrafluoroethylene (PTFE) syringe filters. Chromatographic analysis was performed using an UltiMate 3000 RSLC ultra-high-performance liquid chromatography (UHPLC) system (Thermo Scientific Inc., Waltham, MA, USA). The system consisted of a dual-gradient pump, autosampler, diode array detector (DAD) for positive peak detection set at 325 nm (scanning range 190–400 nm), UV/Vis detector for negative peak detection set at 734 nm, a reaction capillary (100 µL), and two column ovens maintained at 20 °C and 35 °C for the chromatographic column and reaction capillary, respectively. Separation was carried out using an Accucore™ XL C18 column (150 × 4.6 mm, 4 µm) equipped with a C18 guard column (10 × 4 mm) (Thermo Scientific Inc., Waltham, MA, USA) at a flow rate of 0.4 mL/min. The mobile phase consisted of (A) 0.1% formic acid in water and (B) acetonitrile, applied according to the following gradient program: 2.5–10% B (0–10 min), 10% B isocratic (10–20 min), 10–30% B (20–30 min), 30–60% B (30–35 min), 60–95% B (35–40 min), 95% B isocratic (40–45 min), 95–2.5% B (45–47 min), and 2.5% B isocratic (47–55 min). The 2,2′-azinobis-(3-ethylbenzothiazoline-6-sulfonic acid) (ABTS) radical cation solution was continuously pumped through the reaction capillary at a flow rate of 0.4 mL/min. System control and data acquisition were performed using Chromeleon™ 7 software (Thermo Scientific Inc., Waltham, MA, USA). Quantification of CGA was performed using a five-point calibration curve prepared from mixed standards of 3-caffeoylquinic acid (3-CQA), 4-caffeoylquinic acid (4-CQA), 5-caffeoylquinic acid (5-CQA), 3,4-dicaffeoylquinic acid (3,4-diCQA), 3,5-dicaffeoylquinic acid (3,5-diCQA), and 4,5-dicaffeoylquinic acid (4,5-diCQA) (Ambeed, Buffalo Grove, IL, USA). Unknown compounds were quantified as 5-caffeoylquinic acid (5-CQA) equivalents based on the calibration curve prepared using a 5-CQA standard, as 5-CQA is one of the predominant CGAs occurring in coffee and is commonly used as a reference compound for the semi-quantitative determination of related phenolic compounds. Quantification was performed by comparing the peak areas of unidentified compounds detected at the selected wavelength with the response factor obtained for 5-CQA, assuming similar UV absorption characteristics of structurally related CQA derivatives. The antioxidant activity of individual compounds was expressed as trolox equivalents (TEs) by comparing the area under the negative peak at 734 nm with that obtained for the trolox internal standard.

### 3.6. Caffeine

The caffeine content in coffee infusions was determined using UltiMate 3000 RSLC UHPLC–UV/Vis (Thermo Scientific Inc., Waltham, MA, USA) according to ISO 20481:2008 [54], with minor modifications. Briefly, 10 mL of coffee infusion was used as the analytical sample. Prior to chromatographic analysis, the samples were filtered through 0.22 µm polytetrafluoroethylene (PTFE) syringe filters. Chromatographic separation was performed using an Accucore™ XL C18 column (150 × 4.6 mm, 4 µm) equipped with a guard column (10 × 4 mm) (Thermo Scientific Inc, Waltham, MA, USA). The mobile phase consisted of ultrapure water obtained from a Purelab Quest^®^ water purification system (ELGA LabWater, Lane End, High Wycombe, UK) and LC-grade methanol (Honeywell International Inc., Charlotte, NC, USA) mixed in a volume ratio of 70:30 (*v*/*v*). The flow rate was maintained at 1.0 mL/min, and caffeine detection was carried out at 272 nm. Quantification was performed using a five-point external calibration curve prepared from standard caffeine solutions (Tokyo Chemical Industry Co., Ltd., Tokyo, Japan) in the concentration range of 30–120 mg/L.

### 3.7. Statistical Analysis

Statistical analyses were performed using R software version 4.5.2 [55]. All analyses were conducted in three independent experimental series, each performed in triplicate. Differences in quantitative variables among coffee samples prepared using different brewing methods were assessed using one-way analysis of variance (ANOVA). When statistically significant differences were detected, Fisher’s Least Significant Difference (LSD) post hoc test was applied to identify pairwise differences between samples. The significance level was set at 0.05. Principal component analysis (PCA) was also performed to visualize differences among coffee samples according to the brewing method used.

## 4. Conclusions

The present study demonstrated that the antioxidant functionality of coffee beverages is determined not only by the total phenolic content, but more importantly by the qualitative composition and radical scavenging activity of individual CGAs. The results indicate that brewing conditions substantially influence both the extraction profile and the functional properties of bioactive compounds present in coffee beverages.

Among the evaluated brewing methods, cold brew extraction was associated with the highest preservation of CGA and the most pronounced antioxidant potential. Importantly, these differences remained evident after normalization to the amount of coffee used for brewing, indicating that the observed effects cannot be attributed solely to differences in coffee dose and are strongly influenced by the extraction conditions. These findings highlight the importance of brewing parameters in maintaining functionally active phenolic compounds. The results also confirmed that CQA isomers constitute the major contributors to the antioxidant activity of coffee beverages, whereas the contribution of other CGA derivatives appears to be less pronounced.

Importantly, the study demonstrated that the antioxidant potential of coffee cannot be reliably assessed solely on the basis of TPC estimation; composition is much more important. To get an impression of which compounds need to be prioritized in future quality control, the application of HPLC coupled with online TEAC detection enabled a more comprehensive evaluation of the actual contribution of individual compounds to radical scavenging activity and provided functionally relevant insight into the antioxidant behavior of complex coffee matrices.

Overall, these findings contribute to a better understanding of the relationship between brewing technology, CGA composition, and antioxidant functionality in coffee beverages and may support future studies focused on optimizing coffee preparation methods to maximize the retention of bioactive compounds and their potential health-promoting properties.

## Figures and Tables

**Table 1:** Physicochemical characteristics and selected antioxidant-related parameters of coffee beverages prepared using different brewing methods; Americano (A), French press (FP), V60 and cold brew (CB).

Parameter	Brewing Method	Mean ± SD	*p*
Titratable acidity (°)	French press	21.33 ± 0.76	*p* < 0.001 * CB > FP > A > V60
	V60	3.50 ± 0.50	
	Cold brew	34.83 ± 0.29	
	Americano	6.17 ± 0.29	
Caffeine (mg/L)	French press	759.20 ± 1.72	*p* < 0.001 * CB > FP > A > V60
	V60	217.57 ± 5.05	
	Cold brew	939.28 ± 10.29	
	Americano	321.06 ± 6.95	
DPPH radical scavenging activity (%)	French press	19.85 ± 0.27	*p* < 0.001 * CB > FP > A > V60
	V60	15.31 ± 0.20	
	Cold brew	30.74 ± 0.35	
	Americano	16.02 ± 0.20	
TPC (mg GAE/L)	French press	1039.51 ± 9.85	*p* < 0.001 * FP > CB > V60 > A
	V60	654.51 ± 4.53	
	Cold brew	752.45 ± 6.15	
	Americano	620.74 ± 5.85	

*—indicates statistically significant difference (*p* < 0.05); SD—standard deviation.

**Table 2:** Contribution of chlorogenic acids and major mono-caffeoylquinic acid isomers to the antioxidant potential of coffee beverages prepared using different brewing methods.

Brewing Method	Cold Brew	French Press	Americano	V60
Total antioxidant potential − all compounds (µmol TE/L) *	185.40	135.94	50.43	36.29
Total antioxidant potential − all CGAs (µmol TE/L) *	162.84	117.89	42.97	30.82
Contribution of CGAs to total antioxidant potential (%)	87.83	86.72	85.20	84.92
Total antioxidant potential − 3-CQA + 4-CQA + 5-CQA (µmol TE/L) *	127.73	91.62	31.77	22.64
Contribution of 3-CQA + 4-CQA + 5-CQA to total antioxidant potential (%)	68.89	67.40	63.00	62.39
Contribution of 3-CQA + 4-CQA + 5-CQA to the antioxidant potential of all CGAs (%)	78.44	77.72	73.94	73.46

*—antioxidant activity is expressed as µmol of trolox equivalent per liter of beverage.

## Data Availability

The data presented in this study are available on request from the corresponding author due to ongoing research activities and planned future publications based on the dataset.

## Supplementary Materials

The following supporting information can be downloaded at: https://www.mdpi.com/article/10.3390/molecules31122163/s1, Table S1: Results of the analysis of coffee beverages depending on the different brewing methods; Americano (A), French Press (FP), V60 and Cold Brew (CB), expressed per gram of ground coffee used for brewing.
