# Supplementary material for: Brewing-Method-Dependent Changes in the Bioactive Compound Profile and Antioxidant Potential of Coffee Beverages
Source: Molecules. 2026 Jun 19;31(12):2163. doi: 10.3390/molecules31122163 (PMC13304859; doi:10.3390/molecules31122163)
Supplement: Supplementary file 1 [file molecules-31-02163-s001.zip › molecules-4369148-supplementary.pdf]

Table S1. Results of the analysis of coffee beverages depending on the different brewing methods; Americano (A), French Press (FP), V60 and Cold Brew (CB), expressed per gram of ground coffee used for brewing

Parameter	Brewing method	Mean±SD	p
Titratable acidity (°)	Cold brew	34.83±0.29	p<0.001 * CB>FP>A>V60
	French Press	21.33±0.76	
	Americano	6.17±0.29	
	V60	3.5±0.5	
Caffeine (mg/g)	Cold brew	12.52±0.14	p<0.001 * FP>CB>A>V60
	French Press	13.29±0.03	
	Americano	10.18±0.22	
	V60	4.35±0.1	
DPPH radical scavenging activity (%)	Cold brew	30.74±0.35	p<0.001 * CB>FP>A>V60
	French Press	19.85±0.27	
	Americano	16.02±0.2	
	V60	15.31±0.2	
TPC (mg GAE /g)	Cold brew	10.03±0.08	p<0.001 * A>FP>V60>CB
	French Press	18.19±0	
	Americano	19.67±0.19	
	V60	13.09±0	
3-CQA (mg/g)	Cold brew	2.19±0.02	p<0.001 * FP>CB>A>V60
	French Press	2.21±0	
	Americano	1.45±0	
	V60	0.66±0.01	
5-CQA (mg/g)	Cold brew	4.06±0.02	p<0.001 * CB>FP>A>V60
	French Press	3.86±0.01	
	Americano	2.42±0.01	
	V60	1.03±0.01	
4-CQA (mg/g)	Cold brew	6.15±0.04	p<0.001 * CB>FP>A>V60
	French Press	6.04±0.01	
	Americano	4.13±0.02	
	V60	1.85±0.04	
3,4-diCQA (mg/g)	Cold brew	0.11±0	p<0.001 * A>FP>V60>CB
	French Press	0.22±0	
	Americano	0.26±0	
	V60	0.15±0	
3,5-diCQA (mg/g)	Cold brew	0.18±0	p<0.001 * A,FP>CB>V60
	French Press	0.23±0	
	Americano	0.25±0.02	
	V60	0.15±0	

Parameter	Brewing method	Mean±SD	p
4,5-diCQA (mg/g)	Cold brew	0.32±0	$p<0.001$ * A>V60>FP>CB
	French Press	0.43±0	
	Americano	0.75±0.02	
	V60	0.48±0	
Total content of 3-CQA, 4-CQA, 5-CQA (mg/g)	Cold brew	12.41±0.04	$p<0.001$ * CB>FP>A>V60
	French Press	12.11±0.02	
	Americano	8±0.03	
	V60	3.55±0.05	
Unknown compound 1 (mg 5-CQA eq./g)	Cold brew	0.61±0.01	$p<0.001$ * CB>FP>A>V60
	French Press	0.59±0	
	Americano	0.4±0	
	V60	0.18±0	
Unknown compound 2 (mg 5-CQA eq./g)	Cold brew	0.44±0	$p<0.001$ * CB>FP>A>V60
	French Press	0.39±0	
	Americano	0.23±0.01	
	V60	0.1±0	
Unknown compound 3 (mg 5-CQA eq./g)	Cold brew	0.4±0	$p<0.001$ * FP>CB>A>V60
	French Press	0.47±0	
	Americano	0.3±0	
	V60	0.13±0	
Unknown compound 4 (mg 5-CQA eq./g)	Cold brew	0.14±0	$p<0.001$ * FP>CB>A>V60
	French Press	0.19±0	
	Americano	0.13±0	
	V60	0.06±0	
Unknown compound 5 (mg 5-CQA eq./g)	Cold brew	0.48±0.01	$p<0.001$ * FP>CB>A>V60
	French Press	0.74±0.01	
	Americano	0.4±0	
	V60	0.19±0.01	
Unknown compound 6 (mg 5-CQA eq./g)	Cold brew	0.36±0	$p<0.001$ * FP>CB>A>V60
	French Press	0.5±0	
	Americano	0.27±0.01	
	V60	0.13±0	
3-CQA (mg 5-CQA eq./g)	Cold brew	2.47±0.04	$p<0.001$ * FP>CB>A>V60
	French Press	2.53±0	
	Americano	1.68±0	
	V60	0.77±0.01	
5-CQA (mg 5-CQA eq./g)	Cold brew	4.06±0.02	$p<0.001$ * CB>FP>A>V60
	French Press	3.86±0.01	
	Americano	2.42±0.01	

Parameter	Brewing method	Mean±SD	p
	V60	1.03±0.01	
4-CQA (mg 5-CQA eq./g)	Cold brew	2.56±0.03	p<0.001 * FP,CB>A>V60
	French Press	2.6±0.11	
	Americano	1.68±0.01	
	V60	0.75±0.04	
3,4-diCQA (mg 5-CQA eq./g)	Cold brew	0.04±0	p<0.001 * FP>A>V60>CB
	French Press	0.18±0	
	Americano	0.11±0	
	V60	0.05±0	
3,5-diCQA (mg 5-CQA eq./g)	Cold brew	0.14±0	p<0.001 * FP>CB>A>V60
	French Press	0.17±0	
	Americano	0.08±0.03	
	V60	0.04±0	
4,5-diCQA (mg 5-CQA eq./g)	Cold brew	0.02±0	p=0.272
	French Press	0.03±0	
	Americano	0.04±0.02	
	V60	0.03±0	
Total content of 3-CQA, 4-CQA, 5-CQA (mg 5-CQA eq./g)	Cold brew	9.1±0.08	p<0.001 * CB,FP>A>V60
	French Press	8.99±0.12	
	Americano	5.78±0.02	
	V60	2.56±0.05	
Total antioxidant potential of all compounds (μmol TE/g)**	Cold brew	2.47±0.1	p<0.001 * CB,FP>A>V60
	French Press	2.38±0.08	
	Americano	1.6±0.1	
	V60	0.73±0.05	
Antioxidant potential of the total CGA (identified and unidentified) (μmol TE/g) **	Cold brew	2.17±0.1	p<0.001 * CB,FP>A>V60
	French Press	2.06±0.09	
	Americano	1.36±0.08	
	V60	0.62±0.04	
Antioxidant potential of total 3-CQA, 4-CQA and 5-CQA (μmol TE/g) **	Cold brew	1.7±0.1	p<0.001 * CB,FP>A>V60
	French Press	1.6±0.06	
	Americano	1.01±0.05	
	V60	0.45±0.04	

* – indicates statistically significant difference ($P<0.05$);
** – antioxidant activity is expressed as μmol of Trolox equivalent per gram of ground coffee;
SD – standard deviation;
